# Intervertebral Disk Degeneration: The Microenvironment and Tissue Engineering Strategies

**DOI:** 10.3389/fbioe.2021.592118

**Published:** 2021-07-20

**Authors:** Yiming Dou, Xun Sun, Xinlong Ma, Xin Zhao, Qiang Yang

**Affiliations:** ^1^Department of Spine Surgery, Tianjin Hospital, Tianjin University, Tianjin, China; ^2^Department of Biomedical Engineering, The Hong Kong Polytechnic University, Kowloon, Hong Kong

**Keywords:** intervertebral disk degeneration, microenvironment, tissue engineering, scaffold, regeneration, disk herniation, low back pain

## Abstract

Intervertebral disk degeneration (IVDD) is a leading cause of disability. The degeneration is inevitable, and the mechanisms are complex. Current therapeutic strategies mainly focus on the relief of symptoms, not the intrinsic regeneration of the intervertebral disk (IVD). Tissue engineering is a promising strategy for IVDD due to its ability to restore a healthy microenvironment and promote IVD regeneration. This review briefly summarizes the IVD anatomy and composition and then sets out elements of the microenvironment and the interactions. We rationalized different scaffolds based on tissue engineering strategies used recently. To fulfill the complete restoration of a healthy IVD microenvironment, we propose that various tissue engineering strategies should be combined and customized to create personalized therapeutic strategies for each individual.

## Introduction

Low back pain is prevalent in the society and is the number one cause of disability globally; 60–80% of adults experience varying degrees of low back pain (Murray et al., 2012; Hartvigsen et al., 2018). Although the etiology and pathology of low back pain are complex, evidence suggests that low back pain is strongly associated with intervertebral disk degeneration (IVDD) (Luoma et al., 1976). The intervertebral disk (IVD) is a fibrous cartilaginous tissue that connects two adjacent vertebral bodies consisting of three parts: the nucleus pulposus (NP), the annulus fibrosus (AF), and the cartilage endplate (CEP). The NP is a centrally located highly hydrated gel-like tissue surrounded by the AF, comprised of layer-by-layer collagen fiber lamellas. The CEPs are situated at the top and bottom of the vertebral bodies, and they interface the IVDs with the adjacent vertebrae (Lawson and Harfe, 2017). This complex structure plays a vital role in transmitting and absorbing mechanical loading onto the spine and maintaining motor function.

The IVD gradually degenerates due to aging and tissue damage caused by multiple stressors, resulting in vertebral instability, spinal canal stenosis, and spinal segment deformity, causing low back pain and mobility disability (Wang et al., 2016). There are three main events in IVDD: (1) inflammation and catabolic cascades, (2) continuous loss of cells, and (3) decline in cellular functions and anabolic activities (Frapin et al., 2019). NP gradually degenerates under the influence of these events. The AF ruptures as the degree of degeneration increases, while the degenerated NP is extruded and oppresses nerve roots due to abnormal stress, resulting in nerve compression symptoms. The CEP becomes more calcified, which adversely affects the transport of nutrients and metabolites, making it difficult to maintain sagittal stress balance. Inflammatory factors stimulate nerve roots, causing or aggravating pain, and enhancing mobility disability. The deteriorating microenvironment further aggravates IVDD.

Traditional therapeutic strategies (surgical and non-surgical treatments) focus on solving the symptoms. Surgical treatments focus more on physically relieving symptoms at the organ level, such as decompressing nerve roots (discectomy) and removing the degenerated disk (fusion and disk replacement). Non-surgical treatments (non-pharmacological therapy, pharmacological therapy, and interventional therapies) perform limited intervention for the microenvironment through anti-inflammatory, analgesic, and spasmolysant effects. Traditional therapeutic strategies do not promote disk regeneration at the cell level and, therefore, do not reverse the progress of disk degeneration (Harmon et al., 2020). Some patients relapse after treatment, and the disk degeneration might even accelerate the degeneration of adjacent segments (Harper and Klineberg, 2019).

In the early stage of IVDD, the enclosed environment makes it difficult for the external stem cells to exert potential regenerative ability on the NP. In the later stage when the degeneration is severe, the AF will tear, and neovascularization and neoinnervation will occur in the NP. Inflammation caused by newly colonized immunecells further harms the microenvironment and accelerates degeneration. Thus, the deteriorating microenvironment and low self-regenerative ability of the IVD are primary obstacles forregeneration. How to customize suitable regenerative strategies for IVD has become a hot topic of recent research. Tissue engineering strategies combining material science, engineering, and life science will become new clinical therapeutic approaches in the future. Tissue engineering strategies contain three key elements: cells, scaffolds, and biomechanical or biochemical signals (Mhanna and Hasan, 2017). These elements have been applied and extended in IVD tissue engineering. Applications of stem cells in IVD and derivative methods (gene therapy and extracellular vesicle therapy) have shown promising therapeutic potential for IVDD. Illustrating the interactive mechanisms between the components [IVD cells, biological factors, extracellular matrix (ECM) components, and environmental factors] in the IVD microenvironment makes therapeutic strategies more rational. Scaffolds mimic the microstructure of the IVD ECM and provide proper structural support for IVD cells. Scaffolds can carry and release therapeutic biological factors. These findings contribute to IVD tissue engineering strategies. Tissue engineering strategies should be devised, considering the pathological changes of IVDD with the aim to reverse degeneration, namely: (1) cell proliferation should be promoted and cell apoptosis should be inhibited to ensure cell density; (2) the IVD microenvironment should be ameliorated to mitigate cellular stress and inhibit inflammation; and (3) anabolic/catabolic balance should be mediated to ensure the quality and the quantity of the ECM.

## IVD and the Microenvironment

### Anatomy and Composition of the IVD

The IVD is an articular cartilage structure located between the vertebral bodies and accounts for 25–30% of the overall length (height) of the spine. An IVD consists of the NP, the AF, and the CEP. The primary function of the IVD is to provide mechanical support for the vertebral body and allow movement of the spine (flexion, extension, and rotation) (Devereaux, 2007).

The NP is a centrally located highly hydrated gel-like structure consisting of NP cells and the ECM. The cell density in the NP is low (3,000/mm^3^), and the cell types are not completely clear, mainly containing small chondrocyte-like nucleopulpocytes (NPCs) and large vacuolated notochord cells (NCs) (Wang et al., 2014). The ECM of the NP is quite different comparing to that of hyaline cartilage and is mainly synthesized by the NPCs. It contains mostly aggrecan [a large proteoglycan (PG) aggregate], a high ratio of type II/type I collagen, hyaluronic acid (HA), and secondary components (type IX/VI/V collagen and small proteoglycans). PGs are widely found in cartilage, the brain, IVDs, tendons, and corneal tissues. They provide viscoelastic properties, retain water, maintain osmotic pressure, and arrange collagen tissue. Aggrecan in the IVD contains more keratin sulfated (a highly hydrated sulphated glycosaminoglycan). Thus, it provides more hydration ability (Iozzo and Schaefer, 2015; Frapin et al., 2019; Harmon et al., 2020). HA is a widely expressed glycosaminoglycan (GAG) located in the ECM, intracellular environment, and the cell surface that interacts with specific proteins, such as aggrecan, versican, lymphatic vessel endothelial HA receptor-1, tumor necrosis factor (TNF)-inducible gene-6 protein, and thrombospondin as well as membrane receptors, such as CD44, HA-mediated motility receptor, and Toll-like receptor 4/2. Thus, it plays a role in morphogenesis, cell migration, cell survival, apoptosis, inflammation, and tumorigenesis (Dicker et al., 2014). HA binds to PGs to form aggrecan, which highly hydrates NP and generates a hydrostatic pressure to effectively absorb stress, reduce vibration, and maintain the osmotic pressure and disk height of healthy IVDs (Raj, 2008; Brown et al., 2012; Wang et al., 2014). A small part of small leucine-rich proteoglycans (SLRPs) in PGs, which mediate tissue order, cell proliferation, matrix adhesion, and the responses between cell and biological factors, is an important signal transduction factor and receptor for the development, morphogenesis, and immunization activities of IVDs. Recent research has reported that SLRPs are related to IVDD, where the asporin gene in the SLRP family rapidly upregulates after the age of 22. SLRPs also exhibit an increased expression in degenerated IVDs (Song et al., 2008; Gruber et al., 2009). Type II collagen is the most critical collagen in the NP for forming irregular networks to support PGs and water. The distinctive arrangement equalizes the NP stress in different directions and, together with water, makes the NP elastic (Colombier et al., 2014). As IVD degeneration progresses, type II collagen is gradually replaced by low-elasticity type I collagen, and the fibrotic NP gradually loses its biomechanical function.

The AF is comprised of 15–25 layers of angle-ply collagen fiber lamellas containing PGs, arranged in concentric circles outside the NP (Chu et al., 2018), and contains AF cells (AFCs) and ECM. The AF is divided into the outer AF and the inner AF. The outer AF mainly consists of dense and organized type I collagen. Thus, it has robust tensile strength, while the inner AF contains a lower ratio of type I collagen as the transition zone between the AF and NP. The density of AFCs is about 9,000/mm^3^ and mainly consists of fibroblast cells (Raj, 2008). NP generates a hydrostatic pressure when IVDs are under axial stress and releases fluid shear stress to the AF. The multi-lamellated AF structure effectively converts the axial stress to interlamellar stress and produces annular stress to resist it (Liu et al., 2001; Iu et al., 2017). The AF can withstand compression and tensile stress during movement of IVDs.

The CEP is a hyaline cartilage structure that connects the vertebral bodies to the IVD. The CEP is comprised of hyaline cartilage cells and chondrocytes that produce PGs and type II collagen to transport nutrients and metabolites to the IVD, which is avascular tissue. The blood supply ends in the CEP, making the CEP a critical transportation junction. The CEP also bears the stress from the IVD to protect the vertebral bodies (McFadden and Taylor, 1989; Roberts et al., 1989; Fontana et al., 2015; Frapin et al., 2019).

As the core of this structure, the stable and enclosed microenvironment of the NP guarantees the expression of the ECM, which supports and separates the vertebrae, absorbs shock, permits transient compression, and allows for movement. NPCs also affect the expression of the AFC ECM. A healthy ECM (mainly type 1 collagen in the outer AF) is comprised of dense AF lamella, which provides protection for the NP microenvironment. Besides bearing sagittal stress, the CEP is the sole pathway of metabolite exchange for the avascular NP. These three parts together form a unique anatomical structure, which maintains homeostasis of the IVD microenvironment in unity and maintains healthy IVD function.

### Physiological Microenvironment

The IVD is in a unique microenvironment: avascular, hypoxic, hyperosmotic, acidic, and with low diffusion of metabolites and restricted by biomechanics (Roberts et al., 1996; Bartels et al., 1998; Wuertz et al., 2008). During early embryonic development, a rod-like notochord is located in the central area of the embryo and guides the ectoderm folds in on itself over the notochord to form neural tube mesenchymal cells (MSCs) to form vertebral bodies and the AF. NCs are trapped inside and participate in the formation of the NP. NCs are considered to be involved in the regeneration of the NP through cellular dialogue with other cells. NCs disappear in most human adults before the bone matures, but signs of IVDD occur not long after their disappearance (Hunter et al., 2003).

The cellular dialog between NCs and NP cells, which is triggered by multiple biological factors, maintains IVD homeostasis. These biological factors can also mediate the IVD microenvironment.

One of the critical factors mediating cell metabolism is hypoxia-inducible factor (HIF) (Semenza et al., 1994). HIF is a transcription factor that initiates a coordinated cellular cascade in response to a low-oxygen tension environment, including the regulation of numerous enzymes in response to hypoxia (Li et al., 2013). The HIF-1 α-subunits remain stable in hypoxic conditions but rapidly degrade under normoxic conditions. At the same time, HIF-1α and HIF-2α activities are stable in IVD, which illustrates that these two factors are positively related to IVD activity (Wang et al., 1995; Agrawal et al., 2008; Li et al., 2013).

Hypoxia-inducible factor is associated with most cell activities in the IVD. HIF-1α regulates the glycolytic activity of NPCs to ensure an energy supply under hypoxia, which results in the acidic microenvironment in the IVD (Agrawal et al., 2007; Kalson et al., 2008). HIF promotes the synthesis of PGs, type II collagen, and GAGs by direct or indirect pathways (Agrawal et al., 2007; Li et al., 2013; Liu et al., 2017). It also inhibits Fas/FasL-mediated apoptosis of NPCs by inducing the expression of Galectin-3. The expression of HIF-1α increases significantly in degenerated IVDs and is correlated with cell apoptosis (Ha et al., 2006; Li et al., 2013). HIF-2α regulates the expression of aggrecan and type II collagen by regulating the expression of catabolic factors (MMPs-13 and ADAMTS-4). The expression of HIF-2α, MMP-13, and ADAMTS-4 increases significantly, while the expression of aggrecan and type II collagen decreases significantly in degenerated IVDs (Huang et al., 2019), indicating that HIF plays an essential role in maintaining the IVD ECM. The hypoxic environment is destroyed after IVDD occurs; the absence of HIF can further accelerate degeneration (Meng et al., 2018).

Vascular endothelial growth factor (VEGF) is important during vasculogenesis and angiogenesis and mainly targets endothelial cells (Apte et al., 2019). Interestingly, the VEGF protein and its receptors are expressed in the avascular NP (Fujita et al., 2008). VEGF-A has a strong angiogenic activity and specific effects of mitosis and chemotaxis on endothelial cells (Risau, 1997). The expression of VEGF-A can be induced by hypoxia, leading to a local vascular invasion, but, typically, VEGF-A expressed by NPCs will not cause neovascularization. The reason may depend on the inhibition of endothelial cell adhesion and migration by the high aggrecan content in the IVD (Johnson et al., 1976). The complex of VEGF-A and its receptor VEGFR-1 also inhibit NP cell apoptosis (Fujita et al., 2008).

Nucleus pulposus cells stimulate NCs to secrete connective tissue growth factor (CTGF) and sonic hedgehog (Shh) by secreting transforming growth factor (TGF-β). CTGF and Shh together inhibit the expression of MMPs and stimulate the expression of tissue inhibitors of metalloproteinases (TIMPs), which inhibit MMPs, thereby suppressing the degradation of the ECM (Erwin et al., 2006; Erwin, 2008; Frapin et al., 2019). CTGF and Shh also stimulate the anabolism of the NP ECM and inhibit NP neovascularization and apoptosis by inhibiting VEGF, interleukin (IL)-6, and IL-8. Shh also promotes the proliferation of AF and CEP cells.

The bone morphogenetic protein (BMP) family promotes ECM synthesis by impacting the cellular dialog between NCs and NP cells and regulates the production of MMPs (Leung et al., 2017; Frapin et al., 2019). BMP-2 and osteogenic protein-1 (BMP-7) upregulate the expression of aggrecan and type II collagen, promote the synthesis of GAGs, and concomitantly inhibit the expression of profibrotic genes. BMP-2 and BMP-7 are the most effective factors in the family stimulating the accumulation of PGs, while BMP-4 and growth differentiation factor-5 GDF-5 (BMP-14) stimulate the accumulation of collagen (Zhang et al., 2006; Leung et al., 2017; Li et al., 2017).

The characteristics of high osmotic pressure and high hydration ensure the biomechanical function of the IVD. As a weight-bearing organ, the metabolic and cellular activities of the IVD are closely related to the biomechanical microenvironment (Neidlinger-Wilke et al., 2014). External loads on the spine result in intense pressure on the disk. Intradiscal pressure varies from 0.1 to 2.3 MPa in different locations (Wilke et al., 1999; Neidlinger-Wilke et al., 2014). Severe degeneration can unbalance sagittal/coronal stress, resulting in abnormal stress of degenerated segments (Le Huec et al., 2011). NP cells in the IVD are mainly under extensive hydrostatic pressure, AF cells are under tensile strain, and the CEP is under compression due to its location at the interface (Le Maitre et al., 2004; Setton and Chen, 2006). The cytoskeleton of the IVD cells responds to the mechanical microenvironment. High osmotic pressure has positive effects on metabolic activity and matrix gene expression by IVD cells, and changes in hydrostatic pressure affect the synthesis of PGs by regulating the production of nitric oxide (Liu et al., 2001).

**FIGURE 1:**
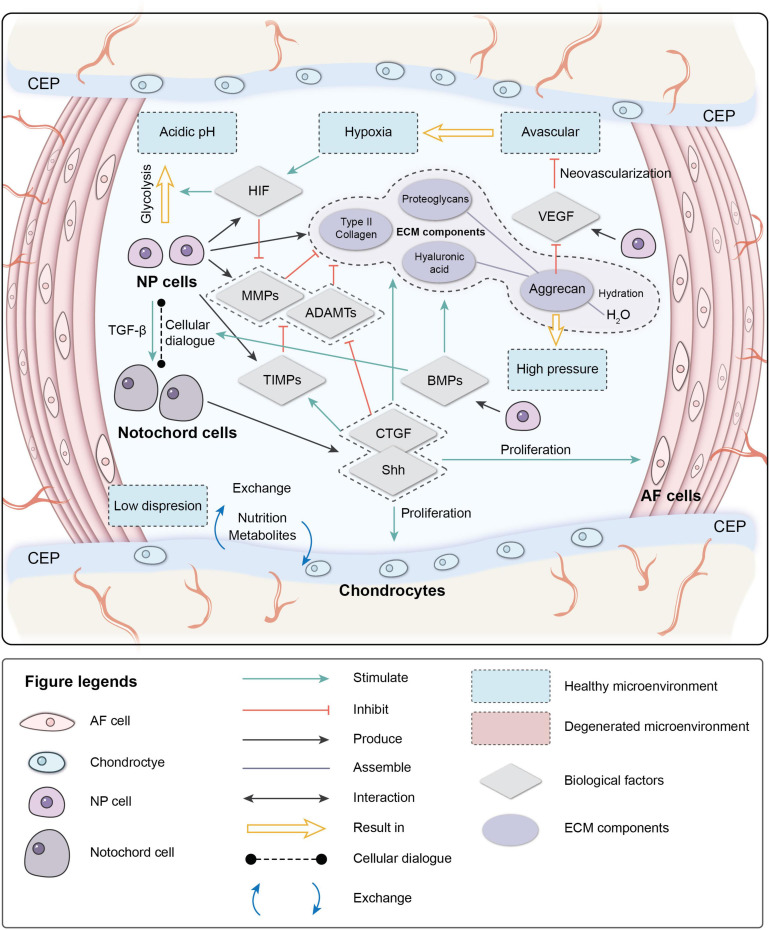
Physiological microenvironment of intervertebral disk (IVD). AF, annulus fibrosus; NP, nucleus pulposus; CEP, cartilage endplate; ECM, extracellular matrix; HIF, hypoxia-inducible factor; MMPs, metalloproteinases; ADAMTs, metalloproteinase with thrombospondin motifs; TIMPs, tissue inhibitors of metalloproteinases; CTGF, connective tissue growth factor; Shh, sonic hedgehog; BMPs, bone morphogenetic protein; VEGF, vascular endothelial growth factor.

Interactions between different components in the IVD microenvironment maintain the homeostasis of the microenvironment (Figure 1). Due to aging, impaired disk function, loss of cells, and imbalance of ECM anabolism/catabolism, the homeostasis of the microenvironment breaks down, eventually resulting in pathological IVDD.

### Pathological Environment

**FIGURE 2:**
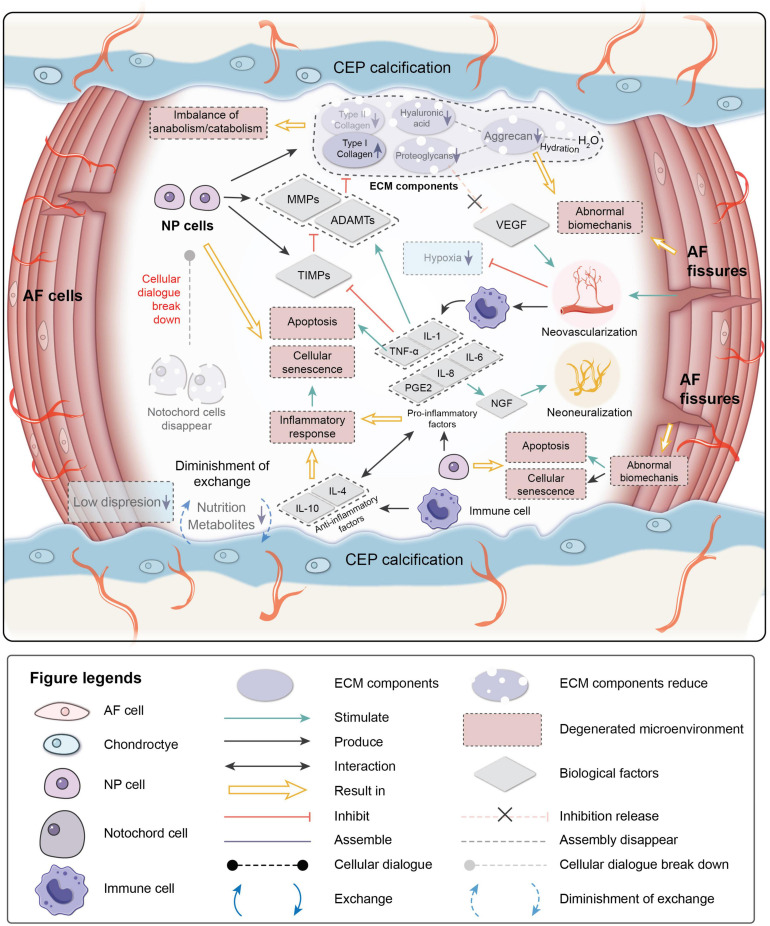
Pathological microenvironment of intervertebral disk (IVD). AF, annulus fibrosus; NP, nucleus pulposus; CEP, cartilage endplate. ECM, extracellular matrix; MMPs, metalloproteinases; ADAMTs, metalloproteinase with thrombospondin motifs; TIMPs, tissue inhibitors of metalloproteinases; VEGF, vascular endothelial growth factor; NGF, nerve growth factor; IL-1,4,6,8,10, interleukin-1,4,6,8,10; TNF-a, tumor necrosis factor; PGE2, prostaglandin E2.

The degenerative mechanisms are very complex and are related tovarious causes, such as age, genetics, the microenvironment, and biomechanics (Frapin et al., 2019). Early degeneration may be asymptomatic. Signs may be detectable by radiography, while the reduction in water content of NP can be visualized by magnetic resonance imaging due to the reduced synthesis of PGs and reduced disk height of the IVD on a computed tomography scan (Modic et al., 1988). Four main changes occur during degeneration: (1) cell senescence, (2) imbalance of ECM anabolism/catabolism, (3) inflammatory microenvironment, and (4) abnormal biomechanics (Figure 2).

#### Cell Senescence

Cellular senescence, which is a fundamental mechanism that mediates age-related dysfunctions and chronic diseases, accumulates in human, bovine, and rat degenerative IVDs during aging (Roberts et al., 2006; Gruber et al., 2007; Kim et al., 2009; Muñoz-Espín and Serrano, 2014; van Deursen, 2014; Sharpless and Sherr, 2015). Unlike apoptosis, senescent cells are metabolically viable and arrest at the cell cycle transition, cease proliferation, and exhibit an altered expression of various catabolic cytokines and degrading enzymes (Campisi, 2005; Davalos et al., 2010; Muñoz-Espín and Serrano, 2014; van Deursen, 2014; Sharpless and Sherr, 2015). Two intrinsic pathways are related to cellular senescence in IVDs: the p53-p21-RB pathway in a telomere-dependent manner and stress-induced premature senescence that activates the p16INK4a -RB pathway independently of telomere length (Ben-Porath and Weinberg, 2005). A stimulus from the microenvironment can cause damage to IVD cells, resulting in early senescence.

Senescent cells in degenerated disks can form senescent cell clusters, which can cause inflammatory stress by secreting pro-inflammatory cytokines and accelerate the senescence of neighboring cells (Wang et al., 2016). As a result, cellular senescence, impaired self-repair capacity, increased inflammation, and enhanced catabolism gradually lead to deterioration of the microenvironment and cause disorder in the cellular dialogue and an imbalance of anabolism/catabolism.

#### Imbalance of ECM Anabolism/Catabolism

The ECM provides mechanical support for the IVD and is essential for maintaining the relatively avascular and aneural nature of a healthy disk. Proteoglycans, which are a primary component of the ECM, retain water and contribute to the osmotic pressure responsible for NP biomechanical properties. In the early stage of degeneration, proteoglycan content gradually decreases and is a sign of early degeneration. Type I collagen makes IVDs fibrotic, while type II collagen makes IVDs elastic. The proportions of collagen in the disk change with degeneration of the matrix. The absolute quantity of collagen changes little, but the type and distribution of the collagen can be altered. The ratio of collagen type II/type I decreases, and fibronectin content increases with increasing degeneration. As a result, the disk becomes more fibrotic and less elastic (Raj, 2008; Wang et al., 2016).

The balance between anabolism and catabolism is positively related to the IVD microenvironment, and the cellular dialogue, metabolic enzyme activity, and biomechanical changes affect this balance. As the population of NCs decreases with age, the cellular dialogue between NCs and NPCs gradually diminishes. Anabolic activity is mediated by biological factor disorders, while catabolic activity continuously progresses (Freemont, 2009).

MMPs, disintegrin, and metalloproteinase with thrombospondin motifs (ADAMTS) are important catabolic factors in disk degeneration. ADAMTS1, 4, 5, 9, and 15 are aggrecanases that degrade aggrecan, while MMP1, 8, and 13 are collagenases that cleave fibrillar collagen (Weiler et al., 2002; Le Maitre et al., 2004; Bachmeier et al., 2009; Vo et al., 2013). Upregulation of MMP and ADAMTS expression and enzymatic activity is implicated in the destruction of the disk ECM, leading to the development of IVDD (Bachmeier et al., 2009; Pockert et al., 2009; Vo et al., 2013). TIMP, which is a specific inhibitor of MMPs, binds to the active forms of MMPs to suppress catabolic enzyme activity. However, the activities of MMPs, ADAMTS, and TIMP are all regulated by inflammation-related factors, particularly pro-inflammatory factors. PGs in the IVD are proposed as a barrier to vascular and neural ingrowth into the NP (Khan et al., 2017). As PG contents decrease, the inhibiting effect for the neovascularization function of VEGF weakens. Moreover, AF fissures caused by abnormal biomechanics allow neovascularization and neoinnervation from a lack of PG production. As a result, NP is invaded by immune cells transported by new blood vessels. Immune cells respond to the deteriorating microenvironment, eventually leading to inflammation.

#### Inflammatory Microenvironment

Inflammation is an adaptive response triggered by noxious stimuli and conditions, such as infection and tissue injury. Regardless of the cause, inflammation presumably evolved as an adaptive response to restore homeostasis. If the acute inflammatory response fails to eliminate the pathogen, the inflammatory process persists and acquires new characteristics (Majno and Joris, 2004; Medzhitov, 2008). The IVD is an avascular tissue until neovascularization occurs due to degeneration. Crystals and ECM breakdown products may be responsible for causing the IVD inflammatory response (Medzhitov, 2008). When anabolism/catabolism becomes unbalanced, degradation products trigger inflammation. In human IVD cells, HA fragments upregulate the mRNA expression levels of the inflammatory and catabolic genes IL-1β, IL-6, IL-8, cyclooxygenase-2, MMP-1, and MMP-13 (Quero et al., 2013). After neovascularization occurs, immune cells migrate to the NP to respond to the microenvironment. NP embryologically develops in an enclosed structure, which makes NP an avascular and immune-privileged tissue. Hence, immune cells invade when disk degeneration progresses to a certain stage, making IVD inflammation a chronic process. Inflammatory factors play essential roles in mediating IVD homeostasis and degeneration.

TNF-α, IL-1β, and IL-1α are expressed by healthy IVD cells and immune cells (macrophages, monocytes, dendritic cells, B cells, and natural killer cells) (Le Maitre et al., 2005; Richardson et al., 2009; Wang et al., 2013), where they have been observed at increased levels along with other inflammatory mediators in degenerated IVDs (Le Maitre et al., 2005, 2007). TNF-α is a cytokine that stimulates the inflammatory response, induces nerve swelling and neuropathic pain, and promotes cellular apoptosis *via* its cytotoxic effect (Pedersen et al., 2015). IL-1β induces a catabolic response by NP cells at the molecular level (Smith et al., 2011). Together they stimulate the production of MMPs and ADAMTS by NPCs and suppress the expression of TIMPs, resulting in decreased synthesis of aggrecan and type II collagen by NPCs (Frapin et al., 2019). Pro-inflammatory factors are also related to pain. IL-6, IL-8, and prostaglandin E2 synthesis by NPCs stimulate nerve growth factor production, which induces abnormal nerve ingrowth and causes pain (Frapin et al., 2019). IL-6 and IL-8 are both higher in severe sciatica patients, and IL-6 is correlates to low back pain frequency (Khan et al., 2017). A herniated disk impinging on the nerve root is painless in 70% of patients, and it is likely that the secretion of products involved in the inflammation cascade in a torn AF sensitizes the nerve root or increases the number of innervations, thereby causing pain (Sharifi et al., 2015). Anti-inflammatory activity is also part of the IVD microenvironment. IL-4 and IL-10 are anti-inflammatory factors produced by activated macrophages and monocytes. They participate in pro-inflammatory/anti-inflammatory balance and inhibit the synthesis of pro-inflammatory cytokines (Khan et al., 2017).

#### Abnormal Biomechanics

Intervertebral disk degeneration can also be affected by the biomechanical environment. A positive dose-response relationship is observed between cumulative lumbar load and early onset of symptomatic lumbar disk space narrowing: the disk disease onset time of workers with the highest exposure to heavy physical constraints is significantly advanced compared to others (Petit and Roquelaure, 2015). Xing et al. (1976) designed an amputated-leg rat model. Due to the forelimb amputation, the IVDs underwent abnormal mechanical loading. The results of senescence-associated β-galactosidase-positive staining showed that IVD cell senescence accelerates due to the abnormal loading of rat lumbar IVDs (Xing et al., 1976). Constant pressure on the disk can cause degeneration, which may be related to the upregulation of matrix degradation-related enzymes (Yurube et al., 2012; Neidlinger-Wilke et al., 2014). The mechanical load can also induce IVD cell apoptosis through the mitochondrial pathway (Rannou et al., 2004; Kuo et al., 2014).

Briefly, IVDs are continually adapting to changes in the microenvironment from embryogenesis to degeneration under the regulation of numerous factors (Colombier et al., 2014). The avascular and immune-privileged NPs regulate cell activity *via* cellular dialogue (Fontana et al., 2015; Henry et al., 2018). ECM synthesized by IVD cells ensures the biomechanical function of IVD. The CEP ensures transport of nutrients and metabolites (Zhang et al., 2018). When degeneration begins, interactions between components in the microenvironment break down, resulting in cascades of degeneration (Freemont, 2009). Therefore, therapeutic strategies should first consider how to relieve pain and regain IVD function in patients and, furthermore, correct the factors causing degeneration to eventually achieve ideal IVD regeneration and functional restoration.

## Current IVDD Treatments

### Non-surgical Treatments

Intervertebral disk degeneration is inevitable; 60–90% of patients can be treated non-surgically as long as no symptoms occur or mild symptoms that occur do not affect daily life (el Barzouhi et al., 2013; Chiu et al., 2015). Non-surgical treatments include non-pharmacological treatments, pharmacological treatments, and interventional treatments. Non-pharmacological treatments, such as exercise, traction, acupuncture, massage, physical therapy, and spinal manipulation, are applied in daily life, but only for second-line or adjunctive treatment options with insufficient evidence for cure (Kreiner et al., 2014; Foster et al., 2018). More than 50% of patients with IVDD have used drugs. Non-steroidal anti-inflammatory drugs are most applied drugs for pain relief and improved function. Oral glucocorticoids alleviate the inflammation of nerve roots. At the same time, muscle relaxants can be useful to relieve muscle spasms, but these drugs are discouraged for lack of sufficient evidence (Kreiner et al., 2014; Ramaswami et al., 2017; Foster et al., 2018; Benzakour et al., 2019; Lee et al., 2019).

A meta-analysis reported that the therapeutic effect of an epidural injection intervention treatment is better than an intradiscal injection, percutaneous discectomy, traction, physical therapy/exercise, radiofrequency therapy, or chemonucleolysis (Lewis et al., 2015). However, interventional treatments are discouraged in the guidelines (Foster et al., 2018).

The goal of non-surgical treatments is to relieve symptoms and improve function, but they cannot halt the degeneration. Surgical treatments are required when non-surgical treatments are unable to relieve symptoms.

### Surgical Treatments

Mixter and Barr (1934) reported that a tumor in the spinal canal, which causes sciatica, was a herniated NP. Laminectomy, combined with excision of the NP, was performed, and satisfactory results were obtained (Mixter and Barr, 1934). Since then, surgical treatments to treat disk herniation have been developed. Four main types of surgical procedures are used: (1) decompression for neurological problems, (2) fusion to abolish motion at a functional spinal unit, (3) motion preservation/modifying surgery in the form of disk replacement/dynamic fixation devices, and (4) deformity surgery to realign the biomechanics between a large number of functional spinal units (Eisenstein et al., 2020). The purpose of decompression surgery is to relieve pain and numbness caused by nerve compression, and the most common procedure is discectomy.

A discectomy removes disk tissue that oppresses nerve roots in the intervertebral space. An emergency discectomy is required for patients who already have cauda equina syndrome and a new motor disorder. Selective discectomy is required for patients with persistent neurological symptoms that cannot be relieved by non-surgical treatments (Butler and Donnally, 2020).

Discectomy can be open or minimally invasive. Open discectomy is performed in a wide range of operations but with more tissue damage. Minimally invasive discectomy causes less tissue damage, but indications are limited. A meta-analysis showed that the incidence of postoperative complications and reoperations is similar, while less blood loss, shorter operating time, and shorter hospital stay are common after minimally invasive discectomy (Alvi et al., 2018; Li et al., 2018a; Butler and Donnally, 2020). Discectomy relieves nerve root compression, relieves pain while retaining some of the structure, and restores some of the biomechanical functions of the disk. However, discectomy cannot change the deteriorating microenvironment. Therefore, further progression of degeneration cannot be prevented, and postoperative complications may occur (12.5, 13.3, and 10.8% for open microdiscectomy, microendoscopic discectomy, and percutaneous microdiscectomy, respectively) (Shriver et al., 2015). The AF must be broken to perform this surgery, and no new AF tissue is formed, so the opening remains open or is closed with the formation of scar tissue, which might cause reherniation (Sharifi et al., 2015).

Spinal fusion is a classic procedure. Hibbs and Albee treated Pott disease using fusion. Fusion abolishes pain by abolishing the motion of adjacent segments (Hibbs, 1964). Spinal fusion is widely used to treat many spinal diseases and is the gold standard for treating significant, chronic axial low back pain due to IVDD. About 60–65% of lumbar fusion procedures are performed for degenerative disk disease (Lee and Langrana, 2004; Auerbach et al., 2009). Spinal fusion completely removes the IVD and entirely relieves the oppression on the nerve roots, where the degenerated segments become integrated and lose motor function. Although the pain symptoms are relieved, the native IVD microenvironment is destroyed. The motor function is abolished, which may cause long-term complications. The mechanical environment of adjacent segments is affected and may develop into adjacent segmental disease (Lee and Langrana, 1984; Hilibrand and Robbins, 2004; Virk et al., 2014; Hashimoto et al., 2019).

Artificial disk prosthesis replacement surgery is performed to restore disk height, biomechanical structure, and motor function of the IVD and overcomes some deficiencies of spinal fusion. This procedure was first applied in the 1950s. As more and more prostheses have been developed, disk replacement surgery has developed quickly (Guyer and Ohnmeiss, 2003; Zhao et al., 2019). Disk replacement surgery effectively relieves pain and improves the quality of life, but it is not a superior substitute surgery for fusion. The average reoperation rate is 12.1% during follow-up after lumbar disk replacement or cervical disk replacement. Postoperative complications, such as prosthesis failure, infection, adjacent segmental disease, and pain, can occur, yet no evidence demonstrates that the surgical effect of disk replacement is better than fusion (Thavaneswaran and Vandepeer, 2014; Cui et al., 2018; MacDowall et al., 2019). The effect of disk replacement surgery greatly depends on the type of prosthesis and surgical technique of the surgeon. An artificial disk prosthesis replicates the anatomical structure of the IVD and attempts to mimic its biomechanical properties. The risk of adjacent segmental disease is reduced compared with fusion (Findlay et al., 2018). However, it is similar to fusion surgery in that the native IVD microenvironment is destroyed. Further follow-up studies are needed to investigate the long-term efficacy and safety of disk replacement surgery.

Several experimental surgeries have been developed. AF sutures strengthen the intensity of AF; thus, they restrict NP from herniating but cannot prevent the progression of IVDD (Marcolongo et al., 2011). Annuloplasty is a minimally invasive method in which heat produced by electricity or radiofrequency radiation strengthens the collagen fibers and seals fissures in a process similar to tissue soldering (Helm Ii et al., 2017). Nucleoplasty releases the pressure on the outer AF, allowing the disk to return to normal size, thereby decompressing the nerve, with better therapeutic effects than non-surgical treatments. This procedure can be performed in the clinic, and the patient recovers quickly after the procedure, but the indications of this procedure are limited (Eichen et al., 2014; Liliang et al., 2016; de Rooij et al., 2017).

Patients who undergo surgery may have better pain relief, functional improvement, and satisfaction than those who receive non-surgical treatments. Nevertheless, long-term follow-up shows that disability outcomes are similar regardless of which treatment a patient receives (Atlas et al., 2001, 2005), suggesting that current strategies can only alleviate symptoms and that regenerative strategy is key to solving IVDD.

## Tissue Engineering Strategies

Surgical treatments for IVDD have developed rapidly, but the limitations of surgical treatments are becoming apparent. Thus, researchers have turned their attention to tissue engineering strategies. Tissue engineering is “an interdisciplinary field that applies the principles of engineering and life sciences toward the development of biological substitutes that restore, maintain, or improve tissue function or a whole organ,” as defined by Langer and Vacanti (1993). Mizuno et al. (2004) fabricated the first documented IVD scaffold of polyglycolic acid and polylactic acid. The scaffold was seeded with AF cells. NP cells were injected into the center after 1 day. Then, the scaffolds were implanted in the subcutaneous space of the dorsum of athymic mice. The results showed that the engineered disks strongly resembled native IVDs and synthesized similar collagen components as native NP and AF (Mizuno et al., 2004; Hukins, 2005). This attempt was initially intended to identify an alternative strategy for IVD replacement due to its long-term deficiencies. The flaws in that study included the cell source, immunological rejection, and fixation of the scaffold, but that study inspired researchers to try tissue engineering strategies for IVDD. Various strategies have been formulated based on different treatment principles. It is important to note that there are no separate strategies, only those that are focused on in the study.

### Scaffold Strategy: From Natural ECM Mimetic to Multifunctional Platform

Scaffolds were initially designed as cell substrates to mimic the microenvironment where cells live. An ECM scaffold is similar to the native microenvironment of cells and has excellent biocompatibility and immunogenicity, which are beneficial for cell proliferation and metabolic activities. Yang et al. (2008) fabricated a decellularized ECM scaffold derived from human cartilage, and the scaffold successfully generated cartilaginous tissue in nude mice. As the AF is a fibrous cartilage structure and has a similar ECM composition to cartilage, an ECM scaffold might be feasible to regenerate AF. McGuire et al. (2017) fabricated an ECM scaffold made of decellularized pericardial tissue. Collagen patches were obtained from treated pericardial tissue. These patches were assembled into a multi-laminate angle-ply scaffold. This scaffold mimicked the structure of AF, provided similar mechanical support, and supported cell viability, infiltration, and proliferation for bovine AF *in vitro* (McGuire et al., 2017).

Complete AF regeneration requires the recovery of biomechanical and structural properties of healthy AF and the restoration of the biological behavior of resident cells in AF (Driscoll et al., 2013). The alignment and organization of AF cells determine their biomechanical functions. As manufacturing technology develops, fabricating methods, such as electrospinning and 3-D printing, provide the possibility for microstructural scaffolds. Microstructural scaffolds guide the cells to form a specific order and are widely applied in AF tissue engineering due to their unique structure. Ma et al. (2018) developed a hybrid scaffold for AF tissue engineering. This hybrid scaffold consisted of traditional electrospun aligned nanofibrous scaffolds (AFS) as a baseline scaffold and electrospun aligned nanoyarn scaffolds (AYS). Morphological measurements showed that this hybrid scaffold replicates the tensile strength, axial compression, and anisotropic properties of AF tissues to some degree. Mechanical testing demonstrated that the tensile properties of AFS and AYS were qualitatively similar to those of native AF tissue. *In vitro* biocompatibility analyses demonstrated that the AYS and HS yield improved bone marrow mesenchymal stem cell (BMSC) proliferation (Ma et al., 2018). Gluais et al. (2019) fabricated electrospun aligned microfibrous scaffolds that recruit neighboring healthy AF cells to migrate to the scaffold, which promotes cell colonization and proliferation. The results showed that, in addition to numerous dense collagen fibers in the aligned scaffolds, the fibers were arranged in the same direction as the scaffold (Gluais et al., 2019). Kang et al. (2018) combined electrospinning and 3D printing techniques to fabricate a biomimetic biodegradable scaffold that consisted of multi-lamella nano/microfibers. Each lamella contained one layer of aligned electrospun nanofibers and one layer of supporting 3D printed microfibers on each side, which were all aligned in the same direction, and the angle of the fibers between adjacent layers was 60°. The nano/microfibres aligned as native collagen tissue of AF. These results show that these scaffolds can form and integrate collagen fibers (Kang et al., 2018).

However, electrospun scaffolds often face several limitations, including low porosity that restricts uniform cell infiltration and a discrepancy of mechanical properties compared with native AF. To recapitulate the form and function of the complex anatomy of AF, Bhunia et al. (2018) adopted a directional freezing technique to fabricate silk-based multi-layered disk-like angle-ply (±30° of successive layers) bio-artificial scaffolds. The fabricated bio-discs supported the primary AF or human mesenchymal stem cell proliferation, differentiation, and deposition of a sufficient amount of specific ECM *in vitro*. The subcutaneous implantation results showed a negligible immune response (Bhunia et al., 2018).

Three-dimensional printing precisely fabricates the scaffold structure. To generate a scaffold with angle-ply architecture similar to natural AF, Christiani et al. (2019) printed laminar constructs comprised of polycaprolactone (PCL) struts oriented at alternating angles of ±30°. The mechanical characterization results showed that the mechanical properties of the scaffolds were similar to native AF tissue. They also cultured bovine AFCs with smooth-surface PCL and unidirectional channel-etched PCL to further study cell arrangement and ECM deposition, respectively. The SEM micrographs of the scaffolds showed that cells cultured on etched PCL had a tendency to align along the underlying surface, and the alignment of proteins with the underlying surface texture can be observed (Christiani et al., 2019).

However, PCL materials have poor hydrophilicity, have a long degradation time, and lack cell recognition sites. PCL materials bind poorly to surrounding host tissues after implantation *in vivo* (Cheng et al., 2017). Three-dimensional bioprinting achieves precise bionics according to the structure and size of native tissues and organs (Kang et al., 2016). As a novel technology, 3D bioprinting shows great potential and enormous advantages in the repair of IVDs. Sun et al. (2021) fabricated a dual growth-factor-releasing IVD scaffold using 3D bioprinting. They loaded CTGF and transforming growth factor-β3 (TGF-β3) on polydopamine nanospheres (PDA NPs). Then, CTGF@PDA and TGF-β3@PDA were mixed with rat BMSCs as the 3D bioprinting ink. A 3D model of the IVD scaffold was designed using AutoCAD software, and the support structures of CEP and AF were printed using a PCL polymer, while the parts of the model corresponding to AF and NP were printed using 3D bioprinting ink. *In vitro* experiments confirmed that the growth factors were released from the IVD scaffolds in a spatially controlled manner and induced the corresponding BMSCs to differentiate into NP-like cells and AF-like cells. After being implanted subcutaneously into the dorsum of nude mice, the reconstructed IVDs exhibited a zone-specific matrix: primarily type II collagen and glycosaminoglycan in the core zone and type I collagen in the surrounding zone (Sun et al., 2021).

In addition to providing mechanical support and topographic stimulus for cells, scaffolds can also be loaded with therapeutic drugs or cells. Cheng et al. (2011) fabricated an injectable thermosensitive chitosan/gelatin/glycerol phosphate hydrogel as a controlled release system for ferulic acid (FA) delivery. FA is an excellent antioxidant drug. The scaffolds were incorporated with FA, added to Transwells, and incubated with H_2_O_2_-induced NP cells *in vitro*. The results showed that these scaffolds achieved excellent antioxidant properties after loading with FA (Cheng et al., 2011).

Scaffolds are a crucial element of this strategy, as they provide a similar microenvironment for IVD cells by mimicking the structure of native IVDs. The scaffold strategy can also be combined with other strategies, such as delivery of therapeutic drugs or cells.

### Cell Therapy Strategy and Derivative Strategies

#### Cell Therapy Strategy

Cell therapy is a classic strategy. An increasing number of studies have shown the efficacy of therapeutic cells in several IVDD animal models (Hiyama et al., 2008; Jeong et al., 2009; Henriksson et al., 2019). One study reported that stem cells and stem-like cells are found in almost all adult tissues (da Silva Meirelles et al., 2006).

Due to the potential of stem cells to differentiate, replacing damaged cells in target tissues, stem cells are ideal therapeutic cells for IVD tissue engineering (Krause, 2008; Meirelles Lda et al., 2009). Mesenchymal stem cells and induced pluripotent stem cells (iPSCs) are the most widely used in IVD cell therapy (Wei et al., 2014; Vickers et al., 2019). Several studies have demonstrated the ability of BMSCs and adipose-derived stem cells to differentiate into an NP-like phenotype, and *in vivo* studies have demonstrated the ability of implanted MSCs to enhance matrix production, particularly GAG synthesis, and increase disk height and hydration (Richardson et al., 2006; Gantenbein-Ritter et al., 2011; Stoyanov et al., 2011; Clarke et al., 2014). iPSCs differentiate into NCs or NP-like cells and reduce IVDD after transplantation *in vivo* (Sheyn et al., 2019; Xia K. et al., 2019).

Researchers have attempted different methods to transport therapeutic cells to diseased areas, such as direct injection of therapeutic cells or loading of cells onto scaffolds. Hiyama et al. (2008), Jeong et al. (2009), and Henriksson et al. (2019) injected human MSCs into rat, dog, and pig disk degeneration models, respectively. The results showed that transplantation of human MSCs has a positive repair effect on the xenogeneic animal disk degeneration model. Sheyn et al. (2019) induced human iPSCs to differentiate into notochordal cells *in vitro* and proved their regenerative capacity *in vivo* in an annular puncture pig model.

However, direct injection by needle puncture causes damage to the AF, and implanted cells could leak out through annular fissures. AF damage can lead to further degeneration and an increased risk of disk herniation (Oehme et al., 2015). Scaffolds could be pivotal to provide transplanted cells with a supportive environment. Bhunia et al. (2018) seeded MSCs on AF structure-like scaffolds and reported ideal results as mentioned above. GDF-5 inhibits IVDD and promotes NP-like differentiation of stem cells (Taylor et al., 2011). However, such a factor could require multiple injections due to its short life *in vivo* (Jin et al., 2016). Xia C. et al. (2019) generated polymeric gelatin microspheres, which can release growth and differentiation factor-5 and act as a cell delivery vehicle for iPSC-derived NP-like cells. Then, they injected these cell-seeded polymeric microspheres into rat coccygeal IVDs, and the results indicated that disk height was recovered, water content was increased, and the NP ECM was partially restored (Xia K. et al., 2019). This study utilized growth factors to enhance the therapeutic efficacy of cells and prolong the life of growth factors *via* a polymeric gelatin microsphere as a sustained release platform. Multiple strategies complementing each other will become more important in IVD tissue engineering.

Recent studies have also revealed the existence of endogenous stem/progenitor cells in the IVD (Hu et al., 2018). Ying et al. (2019) found that stromal cell-derived factor-1α expression is higher in the degenerative IVD microenvironment and showed a positive effect on enhancing the proliferation and recruitment of endogenous NP-derived stem cells into IVDs *in vitro* and *in vivo*. These cells might be a promising source for cell therapy.

Cell-based therapies have the advantages of modulating inflammation and concomitantly affecting the remodeling process, without presenting toxicity or immunosuppression (Lopes-Pacheco et al., 2016). These properties make cell therapy an exceptionally advantageous therapeutic approach for IVD tissue engineering. Nevertheless, various complications occur with stem cells, including tumorigenesis and immune reactions. Certain cases of tumorigenesis and immune reaction of iPSCs and embryonic stem cells (which are also a cell source for iPSCs) have been reported, and the application risks are always discussed (Pera et al., 2000; Taylor et al., 2011; Lee et al., 2013; Kim, 2014; Jin et al., 2016). Compared to iPSCs and embryonic stem cells, MSCs seem to be safer, as no case of tumorigenesis has been reported after MSC transplantation *in vivo* (Oryan et al., 2017).

Although the transplantation of stem cells may have risks, their efficacy cannot be denied. Risks may be hedged by enhancing the immune compatibility of stem cells. Immune rejection is caused by HLA mismatching. Xu et al. (2019) generated HLA pseudo-homozygous iPSCs through CRISPR-Cas9, with allele-specific editing of HLA heterozygous iPSCs and HLA-C-retained iPSCs, which evade T and NK cells *in vitro* and *in vivo*. HLA-C-retained iPSCs combined with HLA-class II knockout are immunologically compatible with >90% of the world’s population, greatly facilitating iPSC-based regenerative medicine applications (Xu et al., 2019). Cell strategies have potential in tissue engineering, but improving safety and avoiding risks should be given more attention in future studies.

#### Therapy Strategies Using Biological Factors

Biological factors are promising therapeutic drugs for IVDD as native mediators in the IVD microenvironment since they are key signaling factors in the cellular dialogue. Unlike conventional drugs, biological factors are secreted by IVD cells and have fewer side effects. Therapeutic biological factors should be able to restore the healthy microenvironment of IVD. Therefore, biological factors should have at least one therapeutic function as follows: (1) pro-anabolism/anti-catabolism, (2) anti-inflammation, and (3) regulate cell activity. The application of biological factors is limited by their short life *in vivo.* Thus, biological factors are often used in conjunction with other strategies.

Researchers can customize biological factor scaffolds according to different conditions, with the key concept of restoring the balance of the microenvironment. Several widely used factors are introduced in Table 1.

**TABLE 1:** Several widely used therapeutic biological factors.

Factors	Targets	Scaffolds	Effects	Functions
GDF-5	Rat adipose-derived mesenchymal stem cells(ADSCs) *in vitro* and rat tail puncture model *in vivo*	Gelatin methacryloyl (GelMA) microspheres	(1) Exhibited good mechanical properties, biocompatibilities and enhanced the ADSCs differentiation into NP-like phenotypes *in vitro*. (2) Maintained NP tissue integrity and accelerated the synthesis of ECM *in vivo*.	Pro-anabolism and induce differentiation (Xu et al., 2020)
IGF-1	Human NPCs *in vitro*		IGF-1 protein treatment upregulated the IFG-1R and ER-α expression and promoted NP cell proliferation, collagen-II, and PCNA expression.	Pro-anabolism, promote proliferation and anti-inflammation (Chen R. S. et al., 2020)
SDF-1	Rat BMSCs *in vitro* and rat tail puncture model *in vivo*	Albumin/heparin nanoparticles (BHNPs)	(1) Induce migration of BMSCs *in vitro*. (2) Induce regeneration of annulus fibrosus and nucleus pulposus *in vivo*.	Pro-anabolism and induce migration (Zhang H. et al., 2018)
CCL5	Bovine AFCs and organ-cultured IVDs *in vitro*. Sheep AF rupture model *in vivo*.	Fibrin Gel	(1) Dose-dependent recruitment effect of CCL5 on AF cells were confirmed. (2) In the organ culture study CCL5 did not stimulate homing of AF cells toward the defect sites. (3) The pilot animal study did not show any repair effect of CCL5.	Induce migration (Zhou et al., 2020)
KGN	Human ADSCs *in vitro* and rat tail puncture model *in vivo*	Amphiphilic copolymer PEG-PAPO	(1) Enhanced the viability, autophagic activation (P62, LC3 II), ECM-related transcription factor (SOX9), and ECM (Collagen II, Aggrecan) maintenance in human ADSCs *in vitro*. (2) The injection of PAKM with human ADSCs yielded higher disk height and water content in rats.	Pro-anabolism and induce differentiation (Yu et al., 2021)
BMP-2	Human NPCs and AFCs *in vitro*		BMP-2 antagonized the IL-18 induced upregulation of aggrecan, type II collagen, and SOX6, resulting in reversal of IL-18 mediated disk degeneration.	Anti-catabolism (Ye et al., 2016)
BMP-7	Human NPCs *in vitro*	Self-assembled peptide RADA-KPSS	Attenuate the expression of MMP-3, MMP-9, ADAMTS-4, NF-κB-p65, IL-1, IL-6, and PGE2, promote the accumulation of ECM proteins and suppress apoptosis of NP cells treated with TNF-α *in vitro*	Pro-anabolism/anti-catabolism, anti-apoptosis, and anti-inflammation (Li et al., 2018b)
Anti-TNF-α	Rat lumbar IVD puncture model *in vivo*		Prevents long-term upregulation of intradiscal TNFα	Anti-inflammation (vashwick-Rogler et al., 2018)
Combination of CCL5, TGF-β1, and GDF-5	Human ADSCs and lumbar ovine IVD organ *in vitro*	Pullulan microbeads	(1) Induced Human ADSCs migration *in vitro* (2) The overall NP cellularity, the collagen type II and aggrecan staining intensity, and the Tie2-positive progenitor cell density in the NP were increased at day 28 compared to the control groups.	Pro-anabolism and induce migration (Frapin et al., 2020)

*GDF-5, growth and differentiation factor 5. IGF-1, insulin-like growth factor 1. TGF-β1, transforming growth factor beta1. SDF-1, stromal cell-derived factor-1. CCL5, chemokine receptor type 5. KGN, kartogenin. BMP-2, bone morphogenetic protein 2. BMP-7, bone morphogenetic protein 7. TNF-α, tumor necrosis factor-alpha.*

#### Gene Therapy Strategy

Biological factors intercellularly mediate cell activities, while microRNAs (miRNAs) mediate intracellularly. Gene therapy is a strategy to achieve the durable expression of a therapeutic gene or “transgene” at a level sufficient to ameliorate or cure disease symptoms with minimal adverse events. Two basic strategies have been proposed. An integrating vector is introduced into a precursor or stem cell so the gene is passed to every daughter cell or the gene is delivered in a non-integrating vector to long-lived post-mitotic or slowly dividing cells, ensuring the expression of that gene for the life of the cell (High and Roncarolo, 2019).

Although the application of gene therapy to the IVD has lagged behind other tissues, gene therapy shows excellent potential and safety for IVD tissue engineering. As an encapsulated and avascular tissue, the sealing property of IVD effectively prevents leakage of the disk contents to other sites in the body (Levicoff et al., 2005). Some researchers are using gene-editing techniques, such as CRISPR, to precisely alter DNA sequences or genetically modify immune cells to imbue them with the ability to fight cancer. TNF-α and IL-1β are inflammatory cytokines related to the inflammatory microenvironment in IVDD through TNFR1/IL1R1 signaling. To regulate this signaling, Farhang et al. (2019) produced CRISPR epigenome-edited lentiviral vectors based on TNFR1/IL1R1 targeted screens and delivered the genes into human NPCs by lentiviral transduction. The expression of the editing vectors was confirmed by qRT-PCR. Measurement of NF-κB activity (which is a downstream transcription factor that mediates TNFR1/IL1R1 signaling), apoptosis, and anabolic/catabolic changes in gene expression demonstrated that the lentiviral vectors significantly downregulated TNFR1 and IL1R1 and significantly attenuated the deleterious microenvironment (Farhang et al., 2019).

Gene therapy provides a potentially ideal tool for many diseases by delivering synthetic miRNAs to regulate gene expression (Deverman et al., 2018). However, there are obstacles to delivering miRNAs directly to target tissues due to their inactivation, low transfection efficiency, and short half-life. Chen W. et al. (2020) synthesized agomir, which is cholesterol-, methyl-, and phosphorothioate-modified miRNA, with good stability and enhanced transfection efficiency, in animals. Agomir penetrates the barriers of the cell membrane and tissues *in vivo* to enrich target cells. Agomir874 downregulates the expression of MMPs in NP by mimicking miRNA874. Chen et al. loaded agomir on an injectable polyethylene glycol hydrogel and injected it into a rat IVD. After 8 weeks, the rat IVD was gradually restored to normal height, similar to the healthy group. These results show that agomir874 regulates the balance of synthesis/decomposition of the NP ECM and inhibits IVDD (Chen W. et al., 2020).

Six gene therapy products have been approved since 2016 (High and Roncarolo, 2019). Gene therapy is promising to be performed “one time,” with long-term and high-value therapeutic effects. However, there are still some problems to be solved, such as safety issues and high treatment expense. Gene therapy is aimed at specific targets, which have been studied thoroughly, to regulate cell activities. Thus, gene therapy cannot be used to regulate a series of therapeutic targets or pathways as in cell therapy. Due to the encapsulated microenvironment of the IVD, gene therapy is still promising and not fully explored for IVDD treatment. Studies on safety and therapeutic mechanisms should be conducted in the future.

#### Extracellular Vesicle Therapeutic Strategy

Studies have confirmed that the mechanism of action of MSCs is predominantly paracrine (Lu et al., 2017; Zhu et al., 2020). Almost all types of cells secrete extracellular vesicles (EVs). As the main components of paracrine activity, EVs derived from MSCs achieve regenerative functions. The three main kinds of EVs are exosomes, microvesicles, and apoptotic bodies (El Andaloussi et al., 2013). Exosomes from MSCs have various effects on IVD regeneration, such as antioxidant, anti-inflammatory, anti-apoptosis, and proliferation-promoting effects (El Andaloussi et al., 2013; Lu et al., 2017; Xia C. et al., 2019). Due to the low immunogenicity and high efficiency of exosomes compared to MSCs, exosomes are promising substitutes for MSCs in cell therapy. The mechanism of its regenerative ability remains unclear, but it is very likely to be related to miRNAs. The level of miR-532-5p was observed to be decreased in TNF-α induced apoptotic NPCs but abundant in bone marrow mesenchymal stem cell (BMSC)-derived exosomes. Zhu et al. (2020) demonstrated that exosomes from BMSCs could deliver miR-532-5p and suppress the IVDD *via* targeting RASSF5. Cheng et al. (2018) also reported that exosomes from BMSCs could deliver miR-21, which could activate the PI3K/Akt pathway in apoptotic NPCs, and further inhibit IVDD.

Exosomes were also confirmed to be associated with pathological changes in IVDD. circRNA_0000253 has the maximum upregulation in degenerative NPC exosomes. Song et al. (2020) found that exosomes in NPCs were differentially expressed in degenerative and normal NPCs. The circRNA_0000253 levels notably increased and the miRNA-141-5p levels markedly reduced in degenerative NPCs compared with normal NPCs. Further research confirmed that the circRNA_0000253 could increase IVDD by adsorbing miRNA-141-5p and downregulating SIRT1 *in vivo* and *in vitro* (Song et al., 2020). This study also demonstrated that, in a degenerative microenvironment of IVD, communication between NPCs by secreting exosomes may aggravate degeneration. Utilizing or blocking degeneration-related exosomes might become a promising therapeutic strategy for IVD degeneration.

The components of exosomes should be thoroughly studied. EVs can also be used to carry therapeutic drugs. Dou et al. (2020) fabricated engineered EVs to deliver drugs targeting inflammation. EVs have also been proven to have therapeutic effects on IVDD, but they have not been applied in IVD tissue engineering. The combination of EVs and tissue engineering is prospective. The study of EVs is still a hot research field. As research progresses, EVs will be a promising therapeutic drug or targeted drug delivery system for regenerating IVDs.

## Conclusion

Intervertebral disk degeneration is a complex and common disease, and tissue engineering strategies provide many innovative methods, but most are still in the experimental stage. Surgical and non-surgical treatments are the most effective and safest strategies to relieve pain and regain mobility for patients with IVDD. However, there are obvious limitations in intervertebral discs: toward engineering tissue the current therapeutic strategies, and relief of symptoms do not mean that degeneration has stopped, as IVDs remain in a hostile microenvironment. Regenerative strategies are key to the reversal of degeneration and need to be further studied.

The application of genes, biological factors, and EV are research hotspots. These applications are better substitutes for therapeutic cells, but their long-term viability and efficacy remain uncertain, and the mechanisms are not fully understood. The analysis of active components and illustration of mechanisms should be emphasized in the next stage to achieve precise therapies. Tissue-engineered scaffolds are more like multifunctional platforms, which may be able to simultaneously regulate cellular activities, load, and the controlled release of therapeutic drugs or cells while providing proper structural support, anti-inflammation, and antibiosis. Compared to traditional materials, such as decellularized ECM and classic synthetic materials, manufacturing technology utilizes superior materials to most traditional materials. Although many biomaterials have been proven to be safe and stable, researchers must develop novel materials and adjust their characteristics according to other strategies.

Every therapeutic strategy has a deficiency. Researchers should combine different strategies to restore the healthy IVD microenvironment and try to customize the best treatment strategy for individual patients according to their personalized microenvironments in the future.

## Author Contributions

YD read the manuscript and wrote the draft. XS reviewed and improved the manuscript. QY set up the framework of this review. XM and XZ provided guidance for YD and XS as consultants. YD and XS contributed equally to this work. All the authors contributed to the article and approved the submitted version.

## Conflict of Interest

The authors declare that the research was conducted in the absence of any commercial or financial relationships that could be construed as a potential conflict of interest.
