# Supplementary material for: Intervertebral Disk Degeneration: The Microenvironment and Tissue Engineering Strategies
Source: Front Bioeng Biotechnol. 2021 Jul 20;9:592118. doi: 10.3389/fbioe.2021.592118 (PMC8329559; doi:10.3389/fbioe.2021.592118)
Supplement: Supplementary file 1 [file Table_1.DOCX]

| Category | Structure | [Therapeutic](D:/Program%20Files%20(x86)/Youdao/Dict/8.9.3.0/resultui/html/index.html#/javascript:;) [strategy](D:/Program%20Files%20(x86)/Youdao/Dict/8.9.3.0/resultui/html/index.html#/javascript:;) | Target | Outcomes | Year |
| --- | --- | --- | --- | --- | --- |
| ECM scaffold | Decellularized ECM scaffold derived from human cartilage | Provide an effective three-dimensional environment for the attachment, proliferation and differentiation of BMSCs | [Cartilage](D:/Program%20Files%20(x86)/Youdao/Dict/8.9.3.0/resultui/html/index.html#/javascript:;) [tissue](D:/Program%20Files%20(x86)/Youdao/Dict/8.9.3.0/resultui/html/index.html#/javascript:;) | 1.In vitro: The scaffold could provide an effective three-dimensional environment for the attachment, proliferation and differentiation of BMSCs.  2.In Vivo: The scaffold successfully generated cartilaginous tissue. | Yang et al.  2008 |
| ECM scaffold | Assembled multi-laminate angle-ply collagen patches made of decellularized peri-cardial tissue | Mimic the structure of AF, provide similar mechanical support and support cell viability, infiltration and proliferation | AF | 1.Better biaxial burst strengths than healthy AF.  2.Good cytocompatibility for bovine AF cells. | McGuire et al.  2016 |
| Microstructural scaffold | Biomimetic  biodegradable scaffold consists of multi-lamella nano/microfibers, sharing nano-topography  and micro-porosity | Mimic the structure of AF, organize collagen fiber  formation and integration | AF | 1.Aligned electrospun nano/microfibers of 13 layers，  2.MSCs had normal metabolic activity in the scaffold，  3.Aligned collagen fibers organized in the scaffold and integrated with surrounding native annulus tissue  4. Slowed down disc degeneration. | Kang et al.  2017 |
| Microstructural scaffold | Electrospun-aligned microfibrous | Mimic the native AF lamellae, recruit neighbouring healthy AF cells to repair AF. | AF | 1. No difference was seen between the random and the aligned scaffolds.  2. Numerous and dense collagen fibers in the aligned scaffold  3.Exhibited promising properties for AF defect closure | Gluais et al.  2019 |
| Microstructural scaffold | Silk-based multilayered angle-ply annulus fibrosus  Construct,consisting of concentric layers of lamellar sheets | Mimic the intricacy of AF anatomy, support cell proliferation, differentiation, and ECM deposition to repair AF. | AF | 1. Provided better compressive modulus than native human AF tissue.  2. Good Cytocompatibility for porcine AF cells and huMSCs.  3.Scaffolds seeded with porcine AF cells showed better ECM deposition than with MSCs.  4. Integrity retained more than 4 weeks and few macrophages infiltrated the scaffolds | Bhunia et al.  2018 |
| Microstructural +cellular scaffold | A composite scaffold of in vitro generated NP tissues surrounded by multilamellated AF tissues generated by aligned nanofibrous polycarbonate urethane scaffolds. | To construct a IVD-like structure in vitro to repair IVD defect. | AF+NP | 1.Histological results showed integrated interface between the NP and AF/PU tissue  2.The composite scaffold showed sufficient mechanical strength. | Lu et al.  2017 |
| Microstructural scaffold | Multi-layer PCL scaffolds fabricated by 3D printing | Mimics the structural and biomechanical properties of the native tissue to repair AF. | AF | 1.scaffolds were in opposing angular orientations of ±30°, replicating the angle-ply arrangement of the native AF tissue.  2.The circumferential tensile moduli was similar to native AF tissue while the axial compressive properties exceeded native AF tissue.  3.The scaffolds supported the attachment, prolifera  tion and ECM metabolism of bovine AF cells. | Christiani et al.  2019 |
| Microstructural scaffold | Electrospun-aligned nanoyarn/three dimensional porous nanofibrous hybrid scaffold | Replicate the bio[mechanical](D:/Program%20Files%20(x86)/Youdao/Dict/8.9.3.0/resultui/html/index.html#/javascript:;) [propert](D:/Program%20Files%20(x86)/Youdao/Dict/8.9.3.0/resultui/html/index.html#/javascript:;)y of AF, providing better platform for cell infiltration and cell–scaffold interaction. | AF | 1. More aligned fibre and bigger pore size than aligned fibrous scaffold.  2. Exhibited notable anisotropic mechanical properties.  3.Better proliferation effect of HS and provide infiltration for BMSCs | Ma et al.  2018 |
| Microstructural scaffold | Biomimetic angle-ply multi-lamellar scaffold | Replicates the complex architecture of AF and support AF cells adhesion, proliferation, infifiltration and guide ECM deposition | AF | 1. Compressive modulus of the scaffolds reached 0.24 ± 0.05 MPa [in](D:/Program%20Files%20(x86)/Youdao/Dict/8.9.3.0/resultui/html/index.html#/javascript:;) [the](D:/Program%20Files%20(x86)/Youdao/Dict/8.9.3.0/resultui/html/index.html#/javascript:;) [range](D:/Program%20Files%20(x86)/Youdao/Dict/8.9.3.0/resultui/html/index.html#/javascript:;) from 0.116 to 2.3 MPa of native AF.  2. The scaffolds had favorable biocompatibility for AF cells | Zhang et al  2020 |
| Biological factor+scaffold | Self-assembled peptide RADA-KPSS  scaffold fabricated by conjugating  BMP-7 short active fragment (KPSS) to the C-terminus of RADA16-I | Anti-inflammatory and anti-apoptotic effects by promoting anabolic processes and inhibiting catabolic processes | NP | 1. The RADA-KPSS peptide could  attenuate the expression of MMP-3, MMP-9, and ADAMTS-4, promote accumulation  of ECM proteins, and increase secretion of GAG as compared with the RADA16-I Peptide  2.NF-κB-p65, IL-1, IL-6, and prostaglandin E-2 (PGE2) proteins and decrease cell apoptosis in RADA-KPSS peptide. | Li et al  2018 |
| Composite disc implants | Polyglycolic acid/polylactic acid seeded with AF cells then inject NP cells into the center of the AF scaffold | Invent a scaffold which is more suitable for replacement surgery | AF+NP | 1. First documented IVD scaffold.  2. Engineered discs strongly resembled those of native intervertebral discs.  3. Tissue-engineered AF was rich in type I collagen and NP contained type II collagen, similar to the native disc. | Mizuno et al.  2004 |
| Drug+scaffold | An injectable thermosensitive  chitosan/gelatin/glycerol phosphate (C/G/GP) hydrogel | As a controlled  release system of ferulic acid to inhibit hydrogen peroxide (H2O2)-induced oxidative stress NP cells via its antioxidant property | NP | 1. C/G/GP hydrogel was very suitable for  sustained delivery of FA.  2. Results showed up-regulation of  aggrecan and type II collagen and down-regulation of MMP-3 in mRNA level and decreased the apoptosis of H2O2- induced oxidative stress NP cells | van Dijk et al.  2015 |
| Biological factor+microstructural scaffold | Bioactive microfibrous poly(L-lactide) scaffold loading with (TGF)-β1 | Mimic the ECM three-dimensional environment of AF and promote anabolic activities | AF | 1.greater amount of  glycosaminoglycans and total collagen  2.higher neo-ECM thickness | Vadala et al.  2012 |
| miRNA scaffold | Injectable polyethylene glycol hydrogel loading with modified miRNA | By delivering gene-hydrogel to degenerated NP, | NP | The expression of MMPs was reduced.  And the synthesis/catabolism  balance of ECM was regulated | Chen et al .  2019 |

Table 1. Details of different tissue engineering strategy .
